# Case Report: Complex Treatment Using Vibroacoustic Therapy in a Patient With Co-Infection and COVID-19

**DOI:** 10.3389/fmed.2022.893306

**Published:** 2022-06-07

**Authors:** Assema Zh. Bekniyazova, Assiya Kadralinova, Maiya E. Konkayeva, Aigerim A. Yeltayeva, Aidos K. Konkayev

**Affiliations:** ^1^Department of Anesthesiology and Intensive Care, Astana Medical University, Nur-Sultan, Kazakhstan; ^2^Department of Anesthesiology and Intensive Care, The National Scientific Center of Traumatology and Orthopedics named after Academician N.D. Batpenov, Nur-Sultan, Kazakhstan

**Keywords:** COVID-19, periprosthetic joint infection, vibroacoustic therapy, co-infection, case report

## Abstract

The present report highlights a case of successful treatment of a 59-year-old patient who experienced pain, swelling, hyperemia, the presence of a wound of the right knee joint, impaired function of the right lower limb, weakness, fatigue, and labored breathing. Sepsis was detected in the patient as a result of periprosthetic infection with concomitant severe COVID-19. The patient was admitted to the hospital for 59 days, with 57 days of treatment of the patient at the intensive care unit. A therapy of multiple organ failure involved complex treatment using antiviral and combined antibiotic therapy, taking into account the sensitivity of the pathogen to antibiotics; glucocorticoid therapy; anticoagulant therapy; the concept of non-invasive ventilation; and vibroacoustic pulmonary therapy as a method of physiotherapy as well. An integrated approach using a vibroacoustic device in the therapy of the patient with sepsis due to periprosthetic infection with concomitant coronavirus infection had a positive effect despite the lack of etiological treatment against the COVID-19.

## Introduction

During the pandemic caused by the COVID-19, humankind has faced difficulties in all areas of their lives. Particularly this pandemic situation affected workers in the field of medicine and health. For example, surgeons had to perform surgical procedures for patients with confirmed coronavirus infection who needed immediate treatment (1–3).

Periprosthetic joint infection (PJI) is a severe sequel that occurs in 1–2% of patients with primary arthroplasties. This condition is associated with a high sickness rate and requires complex therapy strategies (4, 5). Patients with the diagnosis of PJI complicated by coronavirus or bacterial co-infection in most cases face an unfavorable outcome of the treatment (6–8).

Vibroacoustic therapy (VAT) is a kind of sound treatment that implicates transiting pure low frequency sine wave vibrancies into the body using an apparatus with coupled speakers (9). VAT has been endorsed for relieving a pain, increasing a circulation and movability of a patient (10). It also has been examined in therapy of such diseases as fibromyalgia (11), cerebral palsy, and Alzheimer’s disease (12).

Both, the present epidemiological situation and the high mortality due to coronavirus infection throughout the world and particularly in Kazakhstan puzzled all medical workers in search of a solution for this issue. The method of vibroacoustic lung therapy is actively used by our Center for treating many respiratory diseases. Based on the results of the treatment, this method has shown a positive effect in patients with coronavirus infection.

However, the effect of VAT in the treatment of various conditions has not been sufficiently studied (13, 14). Hence, the need for studying this method of therapy is crucial in order to get a more efficient treatment of patients with the comorbid background. The present clinical case describes the complex therapy of a patient with the PJI complicated by the COVID-19 viral infection by VAT using a vibroacoustic pulmonary device.

The clinical case deserves close attention because the patient presented in this study had been identified with coronavirus infection, with a Charlson comorbidity index of three points, which is associated with a high risk of mortality (15). Moreover, the patient was also diagnosed with sepsis, multiple organ failure, and disseminated intravascular coagulation as well, which are associated with a high risk of adverse outcomes.

## Case Description

### Patient Information

A 59-year-old male patient was urgently admitted to the intensive care unit of the hospital. At the time of admission, the patient experienced pain, swelling, hyperemia, the presence of a wound on the right knee joint, dysfunction of the right lower limb, weakness, fatigue, as well as labored breathing at the moment of his admission to the hospital. According to the patient, his appetite was reduced with a repeated occurrence of vomiting. Heredity of the patient does not have any hereditary diseases. The patient was disabled due to a knee injury in 1990. Constantly, before the hospitalization the patient took drugs internally: acetylsalicylic acid 75 mg, bisoprolol 5 mg, clopidogrel 75 mg.

From 09.18.2020 to 09.29.2020, the patient was treated at the surgery department of a private clinic with a diagnosis of right-sided post-traumatic gonarthrosis of stage 3. Mixed contracture and pronounced pain syndrome of the right knee joint were also revealed in the patient. Moreover, the main diagnosis of the patient was complicated by hematoma of the postoperative wound of the right knee joint, and contact dermatitis. The echocardiography had indicated a minimal mitral tricuspid regurgitation.

### Clinical Findings

According to the initial checkup, the patient’s visible skin areas were pale gray in color with mild icteric phenomena. Pastosity of the lateral abdominal surfaces and edema of the operated limb were also observed in the patient. The patient’s body temperature at the time of the examination was 38°C. Breathing of the patient was substantive, frequent (up to 24–25 per minute), shallow, and noisy. Blood pressure reached 130/90 mmHg with a pulse of 105–110 beats per minute. Furthermore, the patient’s lips were dry and the tongue was covered with a brown plaque; the abdomen was not swollen and soft on palpation; peristalsis was bugged and weakened at the time of the checkup. In line with the initial checkup, the SOFA quick test was also performed on the patient that had indicated three points and focused on infection. Therefore, sepsis was not excluded from the diagnosis.

### Timeline

**FIGURE 1:**
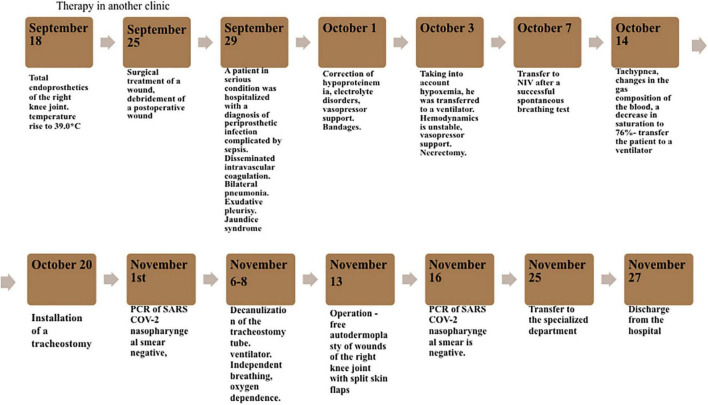
Chronology of the patient’s medical history from the moment of surgery at the private clinic to discharge from the hospital.

Chronology of the patient’s medical history from the moment of surgical procedure at a private clinic to the discharge from the hospital is highlighted in Figure 1.

### Diagnostic Assessment

As a result of routine laboratory tests at the time of the patient’s admission to the hospital, changes in the form of increased markers of inflammation, thrombocytosis, and anemia were revealed. The biochemical blood analysis had detected hypoproteinemia, hypokalemia, hypocalcemia, and hyperbilirubinemia. Hypocoagulation was also indicated in the analysis of the coagulogram. Blood gas analysis also showed a low oxygenation index in the patient’s blood (274.8 mmHg). Additionally, the patient was diagnosed with bilateral pneumonia according to computed tomography (CT) results. Coronavirus infection was not detected by express method COVID-19 – IgM, IgG. CT picture had also indicated moderate hepatomegaly with diffuse fatty hepatosis.

**FIGURE 2:**
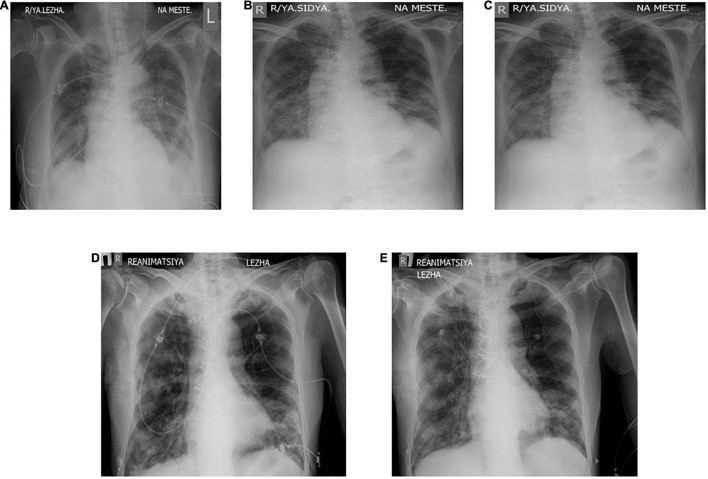
Dynamics of X-ray after the application of VALT: CXR on the 1st day **(A)**, CXR after 18 sessions of VALT **(B)**, CXR on the 16th day (BiPAP: FiO_2_ – 60%, f-18, I/E – 1/2, Pinsp – 25, PEEP – 14) **(C)**, CXR on the 25th day (BiPAP: FiO_2_-40%, f-18, I/E – 1/2, Pinsp-17, PEEP-12) **(D)**, CXR on the 32nd day (BiPAP: FiO_2_-40%, f-20, I/E – 1/2, Pins-20, PEEP 13) **(E)**.

At the time of the admission to the hospital, the patient was diagnosed with bilateral pneumonia, pneumosclerosis, chronic bronchitis, and pneumosclerosis as a condition after coronary artery bypass grafting (CABG; Figure 2A). On the 5th day, the dynamics of the X-ray had shown weakly positive changes in the condition mentioned above (Figure 2B). On the 12th day, X-ray dynamics on bilateral pneumonia were negative (Figure 2C). Furthermore, on the 26th day of the hospital stay, an X-ray picture revealed bilateral polysegmental pneumonia in the stage of incomplete resolution (Figure 2D) with no changes in the X-ray dynamics on the 33rd day (Figure 2E).

The next CT scan of the patient was performed on the 17th day after the hospitalization, and revealed focal darkening as the type of frosted glass with areas of consolidation, involving more than 75% of parenchyma. Pleural effusion had been also noted as a result of the CT. There was also a severe decline in hemodynamics and respiration of the patient, as the oxygenation index at that moment had constituted 108.6 mmHg.

The PCR test results taken from the patient for SARS-CoV-2 detection was positive. A bacteriological study of the material from the tracheostomy tube was also carried out. *Pseudomonas aeruginosa* was detected as a result of the study. Thereafter, such microorganisms as *Morganella morganii, Pseudomonas aeruginosa, Enterobacter aerogenes, Proteus vulgaris* were found in the intubation tube of the intensive care unit on different days of the patient’s stay. In addition, the following types of bacteria were seeded from the area of the patient’s surgical wound: *Escherichia coli* and *Staphylococcus epidermidis* which are the most common pathogens of periprosthetic infections.

Postoperative anemia of moderate severity was also detected in the patient. Sepsis was diagnosed as a condition of the postoperative wound of the right knee joint. Moreover, disseminated intravascular coagulation of blood, and bilateral exudative and interstitial pleurisy was additionally revealed in the patient.

Based on the above mentioned, the preliminary diagnosis of infection and inflammatory reaction caused by endoprosthetics of the right knee joint after the total endoprosthetics of the right knee joint on 09.18.2020 and necrectomy of a postoperative wound on 09.25.2020 was made. In addition, such complications as sepsis, disseminated intravascular coagulation, bilateral pneumonia, bilateral exudative pleurisy, and anemia of moderate severity were also presented in the patient.

Except for the mentioned postoperative complications the following concomitant diseases were revealed in the patient: coronary heart disease, heart failure followed by coronary artery bypass grafting (CABG, surgical procedure was performed on 07.11.2019), as well as chronic bronchitis in remission.

### Therapeutic Intervention

The surgical procedure of total endoprosthetics of the right knee joint was performed on 18.09.2020.

Surgical and postoperative treatment of the wound was carried out on 25.09.2020. The patient received a course of antibacterial therapy, including Vancomycin, hemocorrection (fresh frozen plasma), parenteral nutrition (OliClinomel), and diuretics (Spironolactone, Furosemide).

#### Mechanical Ventilation and Oxygen Therapy

On the 5th day of the hospitalization, the progression of hypoxemia to 58.2 mmHg was transferred to a ventilator in Biphasic positive airway pressure mode with FiO_2_ – 40%, Tinsp – 1.6 s, Fr – 14 per minute, Pinsp – 18 mbar, Pasb – 8 mbar, PEEP – 8 mbar. With these parameters, Exhaled Tidal Volume was provided in 520–540 ml, minute volume – 9.7 l/min. According to the acid-base state analysis, normalization of the level of raO_2_ constituted 95.8 mmHg, raSO_2_ – 45.8 mmHg, pH – 7.403. On the 6th day, the patient was transferred to the constant positive airway pressure mode with FiO_2_ – 35%.

Thereafter, the patient was transferred to non-invasive ventilation on the 8th day after a successful spontaneous breathing test, where active respiratory therapy was performed in the form of non-invasive ventilation and inhalation. Taking into account the increasing tachypnea, changes in the gas composition of the blood, and decrease in the saturation to 76%, it was decided to admit the patient to artificial lung ventilation. On the 21st day, the patient underwent a tracheostomy. Thereafter, on the 37th day, decannulation of the tracheostomy tube was performed. The patient’s breathing was substantive, oxygen dependence, using NIV. Vibroacoustic pulmonary therapy was also performed on the patient every 4 h during the entire stay at the intensive care unit.

#### Antiviral Therapy

After positive results of the PCR test for SARS-CoV-2 detection, Remdesivir was prescribed at 100 mg on the first day of the treatment, followed by 200 mg of I/v h/w dispenser 1 time a day. Remdesivir was subsequently discontinued due to the long QT syndrome according to the electrocardiogram, as the drug’s metabolism and effects are unknown.

#### Anti-infection Therapy

On the first day after the patient’s admission to the hospital, the patient was empirically prescribed an intravenous drip of meropenem 1 g/v through a dispenser, and 20 ml of 0.9% sodium chloride solution at a rate of 40 ml/h, 2 times a day. The next day, this preparation was replaced with 30% lincomycin 600 mg/m intramuscularly. After receiving the results of the analysis of the microbiological study and determining the sensitivity of the isolated cultures of microorganisms, amikacin was added at the dosage of 0.5 g 2 times a day for 3 days. However, after the subsequent microbiological analyses, antibiotics were replaced in the following sequence, depending on the sensitivity of microorganisms: ciprofloxacin 1 g, 2 times a day + ceftazidime 1 g, 2 times a day; moxifloxacin 400 mg, 2 times a day + ertapenem intravenously through a dispenser 1 g, 2 times a day; cefepime intravenously 3 g, 2 times a day. Additionally, the patient received 100 mg of fluconazole daily, enterally 2 times a day.

#### Glucocorticoid Therapy

There also was the administration by the patient of dexamethasone at the dose of 4 mg 2 times a day with the length of administration of 17 days.

#### Anticoagulant Therapy

Enoxaparin at 40 mg 2 times a day and acetylsalicylic acid at 100 mg orally were also administered by the patient.

#### Liquid Volume Management

Transfusion with blood preparations was performed in the patient as well using fresh frozen plasma, washed erythrocytes, and albumin 10% to correct hypoproteinemia.

The assessment of the volemic status was carried out by measuring central venous pressure of the patient, and if necessary, was stimulated with furosemide intravenously at 20 mg 2 times a day. In the case of necessity, up to 100 mg of the preparation was administered using a medication dispenser during the day, under the control of central venous pressure.

#### Nutritional Support

The patient also received nutritional support at the rate of 25–30 kcal/kg/day, and protein provision of 1.2–1.5 g/kg/day.

#### Other

As additional measures, correction of metabolic and electrolyte disorders of the patient was carried out. The patient was prescribed to receive sedation, analgesia, humanistic care, antiarrhythmic, hypotensive drugs, and adrenomimetics under control of blood pressure. Moreover, early-stage physical therapy, including vibroacoustic pulmonary therapy was performed every 4 h.

### Follow-Up and Outcomes

**FIGURE 3:**
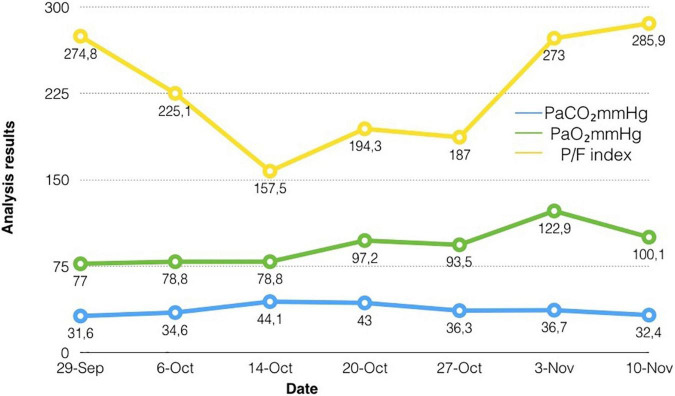
The dynamics of the patient’s respiratory function indicators.

**FIGURE 4:**
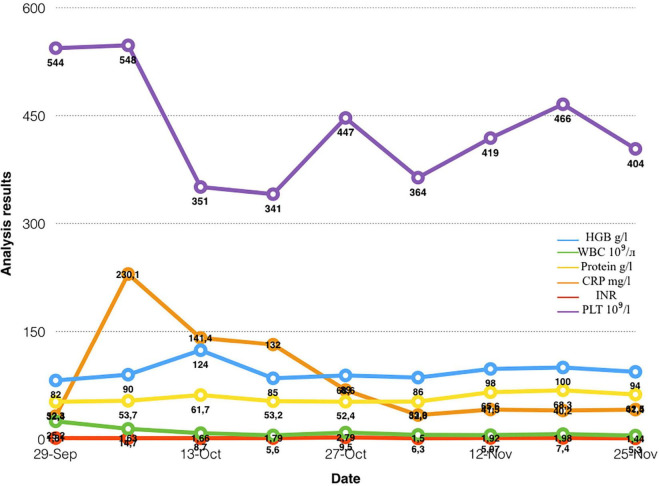
The dynamics of tests from the moment of the patient’s admission to the discharge.

The patient had no contraindications for the conduction of vibroacoustic pulmonary therapy, and no undesirable consequences after the procedure. The tolerance test was carried out with the help of a short application of the device for the vibroacoustic pulmonary therapy for up to 1 min, with the evaluation of parameters of hemodynamics, saturation, and the patient’s sensation. The dynamics of the effect of the vibroacoustic pulmonary therapy were assessed according to the data of peripheral oximetry, blood gas composition, oxygenation index, and radiography of the chest organs. There was a short-term decrease in saturation up to 30 s, associated with active sputum discharge. Clinically, when using the apparatus, we observed an improvement in the drainage function of the bronchi. The drainage effect was also visually recorded during bronchoscopy. The positive dynamics from the treatment were assessed according to the data of peripheral oximetry, blood gas composition, oxygenation index, and radiography of the chest organs dynamics. The implementation of vibroacoustic therapy in the complex treatment of the patient contributed to faster rehabilitation and activation, and as a result, the length of the stay in the intensive care unit was significantly reduced. The dynamics of the patient’s respiratory function indicators are shown in Figure 3. Additionally, the dynamics of tests from the moment of the admission to the hospital to the discharge of the patient are highlighted in Figure 4.

The patient with improved clinical and laboratory data, CT and X-ray diagnostics, negative PCR test for the COVID-19 was transferred to a specialized department in a satisfactory condition, and 2 days later, the patient was discharged from the hospital for rehabilitation treatment at the place of a residence.

## Discussion

As it is known, there is a tendency for unfavorable outcomes in a patient due to the presence of several disorders. Our patient had a comorbid background of the COVID-19 infection and the accompanying pathology of the cardiovascular system. Additionally, the patient had PJI, sepsis, disseminated intravascular coagulation, and multiple organ failures as well. Twenty-three days after the hospitalization, these conditions were complicated by infection with the severe infectious disease – COVID-19. We used complex treatment with vibroacoustic therapy. The treatment of coronavirus infection was carried out in accordance with the protocol “Coronavirus infection – COVID-19,” 10th edition with amendments from 15.07.2020 of the Kazakhstan from 15 July 2020 Protocol No. 106.

To date, with the active spread of coronavirus infection around the world, it is crucial to be wary of the atypical course of other infections, including PJI, since the symptoms of one infection can disguise themselves as symptoms of another infectious disease. The combination of the COVID-19 and PJI has a more severe course and, accordingly, the approach to treatment becomes more complex (4). For this purpose, a multi-system approach to the treatment of this category of patients is required.

One clinical case was found by authors as a result of the literature search on the topic in the PubMed and MEDLINE databases (16). This clinical case describes a patient with comorbid background with COVID-19 infection and PJI, and its management in the operating room.

As it is known, the treatment of patients with concomitant cardiovascular pathology with the background of COVID-19 has its own complications. Pathological processes in this case have a tendency of entangling one another according to the type of “vicious circle” (17).

Physiotherapy is an important and necessary stage in treating such comorbid backgrounds to improve and accelerate the outcomes of the disease (18–20). Vibroacoustic therapy, as one of the methods of physiotherapy, has a beneficial effect on vibration areas by improving blood circulation (9). The method was carried out by our department using the “VibroLUNG” device, which is specially designed for vibroacoustic “massage” of the chest. Due to the use of special emitters, intense exposure is perceived comfortably due to the large coverage area with vibroacoustic emitters. Besides, the effect is transmitted through the air due to the absence of direct contact between the movable membrane and the “irradiated” surface. The device can replace manual methods of percussion and vibration chest massage in the case of respiratory system diseases (21).

Although analysis of the literature found on vibroacoustic lung therapy had not provided extensive information on the following comorbidity, the practical application of the treatment demonstrates positive results in our patients. Conducting vibroacoustic therapy sessions every 3 h in the combination with the main treatment had had a significant effect on lung function and, as a consequence, the outcome of the disease. However, full mechanisms of action of the vibroacoustic apparatus on an organism, namely on lungs, have yet to be studied (9).

The present case shows, that an integral approach to the patient with a severe course of coronavirus infection on the background of comorbidity led to a favorable outcome. Since the COVID-19 is widespread all over the world nowadays, clinicians need to learn more about the treatment of patients with the COVID-19 along with other pathology. We hope that this clinical case will help in providing care to patients with COVID on the background of PJI.

## Patient Perspective

According to the patient, the hardware massage was more pleasant than the manual. Moreover, the vibroacoustic pulmonary therapy via the device had contributed to more relieving cough, and improved general condition and the health of the patient.

## Author’s Note

The authors have read the CARE Checklist (2013), and the manuscript was prepared and revised according to the CARE Checklist (2013).

## Ethics Statement

Ethical review and approval were not required for the study on human participants in accordance with the local legislation and institutional requirements. The patients/participants provided their written informed consent to participate in this study. Written informed consent was obtained from the individual(s) for the publication of any potentially identifiable images or data included in this article.

## Author Contributions

AKo, MK, and AY: conceptualization and organization of the database. AKa: writing draft. AB: review and editing of the manuscript. All the authors issued final approval for the version to be submitted.

## Conflict of Interest

The authors declare that the research was conducted in the absence of any commercial or financial relationships that could be construed as a potential conflict of interest.

## Publisher’s Note

All claims expressed in this article are solely those of the authors and do not necessarily represent those of their affiliated organizations, or those of the publisher, the editors and the reviewers. Any product that may be evaluated in this article, or claim that may be made by its manufacturer, is not guaranteed or endorsed by the publisher.
